# A pilot project on coordination between two levels of care: heart failure unit and programme for prevention and support on discharge (PiSA-IC)/Una experiencia de coordinación entre dos niveles asistenciales: Unidad de Insuficiencia Cardiaca—Programa de prevención y soporte al Alta (PiSA-IC)

**Published:** 2012-05-29

**Authors:** M. Marichal Planas, LL. Garcia Garrido, J. Roure Fernandez, A. Armengou Arxé, S. Valverde Rodríguez, M. González Marcos

**Affiliations:** Girona Primary Care Team, Banyoles, Spain; Dr. Josep Trueta University Hospital of Girona, Girona, Spain; Dr. Josep Trueta University Hospital of Girona, Girona, Spain; Dr. Josep Trueta University Hospital of Girona, Girona, Spain; Dr. Josep Trueta University Hospital of Girona, Girona, Spain; Girona Primary Care Team, Banyoles, Spain

**Keywords:** heart failure, patient education, self-management, multidisciplinary team, case manager, nursing, insuficiencia cardiaca, educación al paciente, automanejo, equipo multidiciplinar, gestor de casos, enfermería

## Introduction

Heart failure (HF) is the third cause of cardiovascular death in Spain, after ischaemic heart disease and cerebrovascular disease [1]. As in other developed countries, HF is the leading cause of hospitalisation of people over 65 years of age and accounts for more than 5% of hospital stays, this corresponding to 70% of the costs associated with HF syndrome [2]. Moreover, the number of admissions due to this condition has increased in recent years in Spain. This is attributable to an increase in the prevalence of the diseases due to population ageing [3]. HF-related healthcare accounts for 2% of the total healthcare budget [2], without taking into account the personal costs of the disease in terms of patient quality of life and early death.

It is important to clinically monitor such patients, since a relatively large number of clinical worsening events can be avoided with a series of check-ups, these having demonstrated their usefulness in decreasing recurrent hospital admissions [4]. In recent years, the action strategies for HF syndrome have changed, mainly in primary care; the great difference is a change in approach due to acceptance that HF is not only an acute disease (as treated to date in hospital settings), but also a chronic clinical syndrome that should be diagnosed early and monitored, with an emphasis on the need for prevention. It has been demonstrated that a multidisciplinary approach across various levels of care and systematic programmes improve patient quality of life and satisfaction as well as decreasing the number of hospital admissions [5].

In our region, with the aim of improving healthcare for this type of patient, we opted for centralising services around the core provider, the Heart Failure Unit—which is hospital based—and at the same time establish a programme for prevention and support on discharge (PiSA), shared with primary care, reaching out to patients in their environment. In the Heart Failure Unit, patients receive multidisciplinary medical and nursing care: they are diagnosed, assessed and treated progressively, according to the course of their condition. At the same time, we initiate and develop their specialised healthcare education. This activity is continued when the patient returns home through the care provided by the case manager of the PiSA programme. These nurses who have received special training are members of the primary and specialised care teams and also know about the patients’ home environments. We carried out a study in order to describe the characteristics of the pilot project on coordination between these two levels of care and to assess the results.

## Methods

This was a descriptive cross-sectional study carried out between June 2006 and May 2009. A total of 208 patients diagnosed with HF were included. The inclusion criteria were: being diagnosed with HF NYHA functional class II, III or IV, independent or dependent with family support, and resident in one of seven basic health areas (geographical zones for the administration of healthcare) in the catchment area of the hospital. Patients with significant cognitive impairment were excluded. Demographic and clinical variables were analysed as well as attendances to the emergency department and hospital admissions.

During hospitalisation, patients were recruited to the programme and then an agreement was reached with regards to their future monitoring through outpatient appointments with the specialist consultant and the hospital clinical nurse. On discharge, patients continued to receive education and their progress was monitored in their own home by the primary care nurse case manager. Communication between the hospital and primary care was maintained using the information technology systems of the Catalan Institute of Health (public provider of healthcare in Catalonia) and by telephone. In addition, patients and relatives were able to contact the PiSA team on a designated telephone number. The intervention carried out in these patients is described in detail in the Heart Failure Unit clinical guidelines. A computer programme was developed for the registration and follow-up of patients as well as recording of the study data.

The centres in the basic health areas of the catchment area of the hospital have patient referral forms to establish a diagnosis, seek advice on management and request further tests (e.g., an ECG).

## Results

The study population had an average age of 65 years and 60% were men. A total of 40.7% of the patients had NYHA functional class III and their mean ejection fraction was 33%. The most common aetiology was ischaemic heart disease (43.7%), followed by hypertensive heart disease (23.2%). During the study period, the number of attendances to the emergency department **decreased by 67%** (mean number of attendances in the pre-intervention period: 1±0.084 vs. in the intervention period 0.33±0.06, p<0.001). Further, hospital admissions were **reduced by 60.27%** (1.4±0.114 vs. 0.56±0.104, p<0.001) and the mean length of hospital stay of patients admitted for HF fell by 60.84% (8.79±0.71 vs. 3.46±0.658 days, p<0.001).

## Conclusions

In our experience, for patients with chronic heart failure, a health care model based on coordination between two levels of health care, ensures consistency in treatment and patient education and improves follow-up, strengthening self-management of the condition. In our region, this type of intervention considerably reduced the number of attendances to emergency departments, hospital admissions and mean length of hospital stays. Early intervention on discharge by the multidisciplinary team across the levels of care means that patients learn about their own condition, recognise when there is clinical worsening and inform the PiSA team, all of this decreasing the overall amount of hospital care required.

## Conference abstract Spanish

## Introducción

La insuficiencia cardiaca (IC) constituye la tercera causa de muerte cardiovascular en España, después de la cardiopatía isquémica y la enfermedad cerebro-vascular [1]. Como en otros países desarrollados, la IC es la primera causa de hospitalización en mayores de 65 años y genera más del 5% de la estancia hospitalaria, lo cual constituye el 70% de los gastos que ocasiona el síndrome de la IC [2]. El número de ingresos por este motivo ha aumentado en los últimos años en nuestro país, debido al propio aumento de la prevalencia de la enfermedad causado por el envejecimiento de la población [3]. La asistencia sanitaria a la IC se estima que representa un 2% del coste sanitario global [2], considerando aparte, el coste personal de esta enfermedad sobre la calidad de vida de la persona y la mortalidad precoz.

Especialmente importante es el seguimiento clínico de estos pacientes, ya que un buen número de desestabilizaciones se podrían prevenir con una serie de controles que han demostrado su efectividad evitando ingresos hospitalarios reiterados [4]. En estos últimos años, se han sucedido cambios en las estrategias de actuación para el síndrome de la IC, principalmente en el ámbito de la atención primaria, la gran novedad está en el cambio del abordaje, es decir, la IC no es tan solo una condición patológica aguda (tratada hasta el momento de manera hospitalaria), sino que estamos ante un síndrome clínico crónico que debemos diagnosticar precozmente, seguir su evolución y principalmente incidir en su prevención. Se ha demostrado que el abordaje interdisciplinar entre diferentes niveles asistenciales y los programas sistemáticos, mejoran la calidad de vida, la satisfacción y disminuyen el número de ingresos hospitalarios [5].

En nuestro entorno, con el afán de mejorar la atención sanitaria de esta población, se apostó por centralizarla en parte en un núcleo básico, la Unidad de Insuficiencia Cardíaca (UIC)—hospitalaria—y creando a la vez, un programa de prevención y soporte al alta (PISA) que comparte aspectos de la atención continuada con Atención Primaria, llegando así al paciente en su ámbito más próximo. En la unidad de insuficiencia cardíaca el paciente recibía una atención interdisciplinar médico-enfermería siendo diagnosticado, valorado y tratado de manera progresiva y adecuada a su evolución. A la vez, también se iniciaba y se progresaba en su educación sanitaria especializada. Estas acciones se prolongaban incluso en el propio domicilio del paciente con la atención que recibía por parte de las enfermeras gestoras de casos del programa PISA. Estas enfermeras formadas de manera especializada, están integradas en el equipo de Atención Primaria y Atención Especializada (UIC), siendo además conocedoras del entorno del paciente. Nuestra experiencia de coordinación entre estos dos niveles asistenciales fue objeto de nuestro estudio que pretendía mostrar la experiencia y analizar sus resultados.

## Metodología

Estudio descriptivo transversal realizado desde junio 2006 hasta mayo del 2009. Se registraron 208 pacientes diagnosticados de IC. Los criterios de inclusión eran: estar diagnosticado de IC en clase funcional (CF) II, III y IV según criterios NYHA; que fuera autónomo o paciente dependiente con soporte familiar; y que perteneciera a las siete Áreas Básicas de Salud de la zona de referencia del Hospital. Los pacientes con deterioro cognitivo evolucionado eran excluidos. Se analizaron variables demográficas y clínicas, así como las atenciones en urgencias e ingresos hospitalarios. Durante el ingreso hospitalario se realiza la inclusión de los pacientes en el programa y se pacta el seguimiento que se lleva a cabo en consultas externas del médico especialista y la enfermera clínica hospitalaria. Al alta, la enfermera gestora de casos de Atención Primaria continúa el proceso educativo y controla su evolución en el propio domicilio. La comunicación entre Hospital y Atención Primaria se realiza mediante los sistemas de información del Instituto Catalán de la Salud (proveedor de servicios sanitarios públicos en Cataluña) y el teléfono móvil de la unidad. También los pacientes y familiares se comunican con el PiSA a través del teléfono móvil asignado. La intervención a realizar en estos pacientes está desarrollada en la guía clínica de la UIC. Se creó un programa informático para registrar los pacientes, hacer el seguimiento y recoger las variables del estudio.

Actualmente, las Áreas Básicas de Salud de la zona de referencia del Hospital disponen de formularios de derivación para establecer el diagnóstico, consultar problemas de manejo y solicitar exploraciones (ecocardiograma).

## Resultados

De la población estudiada el 60% eran hombres, la edad media fue de 65 años. El 40,7% estaban en CF III y con una Fracción de Eyección (FE) media del 33%. La etiología más frecuente era la cardiopatía isquémica (43,7%), seguida de la hipertensiva (23,2%). Durante este periodo, las atenciones en urgencias se **redujeron un 67%** (atención media periodo pre-intervención 1±0,084 vs atención media periodo post-intervención 0,33±0,06 p<0,001). Los ingresos hospitalarios **disminuyeron un 60,27%** (1,4±0,114 vs 0,56±0,104 p<0,001) y la estancia media hospitalaria de los pacientes ingresados por IC se redujo un 60, 84% (8,79±0,71 vs. 3,46±0,658 días p<0,001).

## Conclusiones

En nuestra experiencia, el modelo asistencial basado en la coordinación entre los dos ámbitos asistenciales unifica el tratamiento, la educación y mejora el seguimiento de los pacientes con insuficiencia cardiaca crónica, alcanzando el automanejo de la enfermedad. En nuestro ámbito, este tipo de intervención reduce las atenciones en urgencias, los ingresos hospitalarios y la estancia media. La intervención temprana al alta hospitalaria por parte del equipo interdisciplinar entre niveles asistenciales determina que el paciente conozca su enfermedad, reconozca su agudización y avise al equipo PiSA, todo ello ha hecho disminuir la atención hospitalaria global.
